# Supplementary material for: Growth dynamics and protein-expression of Escherichia coli serotypes O26:H11, O111:H8 and O145:NM in the bovine rumen
Source: PLoS One. 2025 Jun 4;20(6):e0313978. doi: 10.1371/journal.pone.0313978 (PMC12136435; doi:10.1371/journal.pone.0313978)
Supplement: S1 Text — (PDF) [file pone.0313978.s001.pdf]

```r
library(tidyverse)
library(topGO)
library(funfuns)

# iBAQ
PG <- read_tsv('output/protein_groups_cleaned.tsv')
prot_descripts <- read_tsv('output/protein_descriptions.tsv')

uniprot_annotations <- read_tsv('output/pan_0.05_annotations.tsv') %>%
  mutate(accno=From) %>%
  dplyr::select(accno, everything(), -From, -`pH dependence`)

# uniprot_annotations
# PG$Peptides

iBAQ_df <-
  PG %>%
  filter(!is.nan(iBAQ)) %>%
  filter(iBAQ != 0) %>%
  filter(Peptides > 1) %>%
  filter(!(grepl('CON', accno))) %>%
  dplyr::select(accno, contains('iBAQ')) %>%
  transmute(accno,
            iBAQ_L=`iBAQ lactation`,
            iBAQ_M=`iBAQ maintenance`) %>%
  mutate(riBAQ_L=iBAQ_L/sum(iBAQ_L),
         riBAQ_M=iBAQ_M/sum(iBAQ_M)) %>%
  pivot_longer(names_to = c('type', 'condition'), names_sep = '_', values_to
= 'value', cols = -c(accno))

p1 <-
  iBAQ_df %>%
  ggplot(aes(x=condition, y=value, fill=condition)) +
  geom_col(color='black') +
```

```
  facet_wrap(vars(type), scales = 'free') +
  ggtitle('Figure 1: Raw iBAQ and riBAQ normalized intensities')


riBAQ_df <- iBAQ_df %>% filter(type == 'riBAQ')

pseudo_val <- min(riBAQ_df$value[riBAQ_df$value !=0]) / 2

# riBAQ_df %>%
#   filter(value < .0001) %>%
#   ggplot(aes(x=(value))) +
#   geom_histogram(bins = 50)


riBAQ_res <-
  riBAQ_df %>%
  group_by(accno) %>%
  mutate(only_L=value[condition == 'L'] > 0 & value[condition == 'M'] == 0,
         only_M=value[condition == 'M'] > 0 & value[condition == 'L'] == 0,
         both=only_L == only_M) %>%
  ungroup() %>%
  mutate(value=value + pseudo_val) %>%
  group_by(accno) %>%
  summarise(only_L=unique(only_L), only_M=unique(only_M),both=unique(both),
            l2FC=log2(value[condition=='L']/value[condition== 'M']))


# THIS ONE
p2 <-
  riBAQ_res %>%
  ggplot(aes(x=l2FC, fill=both)) +
  geom_histogram(bins=50)+
  lims(x=c(-17,17),
       y=c(0,100)) +
```

```
  annotate(x=9, y=50, geom='label', label='Enriched in Lactation')+
  annotate(x=-9, y=50, geom='label', label='Enriched in Maintenance')+
  geom_vline(xintercept = 0)+
  labs(fill='Detected in both diets',
       y='count') +
  theme(legend.position = 'bottom')+
  ggtitle('Figure 2: Histogram of all log2FoldChange values')


# THIS ONE SIGS
p3 <- riBAQ_res %>%
  filter(abs(l2FC) > 1) %>%
  arrange((l2FC)) %>%
  left_join(prot_descripts) %>%
  ggplot(aes(x=l2FC, fill=both)) +
  annotate(x=9, y=50, geom='label', label='Enriched in Lactation')+
  annotate(x=-9, y=50, geom='label', label='Enriched in Maintenance')+
  geom_vline(xintercept = 0)+
  geom_histogram(bins=50)+
  lims(x=c(-17,17),
       y=c(0,100))+
  labs(fill='Detected in both diets',
       y='count') +
  theme(legend.position = 'bottom')+
  ggtitle('Figure 3: Histogram of log2FoldChange values  > 1')



sig_L_iBAQ <-
  riBAQ_res %>%
  filter(l2FC > 1) %>%
  arrange(desc(l2FC)) %>%
  left_join(prot_descripts)
```

```
up_L_iBAQ <- nrow(sig_L_iBAQ)

sig_M_iBAQ <-
  riBAQ_res %>%
  filter(l2FC < -1) %>%
  arrange(l2FC) %>%
  left_join(prot_descripts)


up_M_iBAQ <- nrow(sig_M_iBAQ)


##
num_not_not_diff <- riBAQ_res %>%
  filter(abs(l2FC) < 1) %>% nrow()

#
num_only_L <- riBAQ_res$only_L %>% sum()
num_only_M <- riBAQ_res$only_M %>% sum()
num_both <- riBAQ_res$both %>% sum()
total <- nrow(riBAQ_res)

# num proteins detected
T1 <- tribble(~category, ~ 'number of proteins',
        'total',total,
        'both diets',num_both,
        'lactation only',num_only_L,
        'maintenance only',num_only_M)


# 'sig' different proteins
T2 <- tribble(~category, ~'number of proteins',
        'not different', num_not_not_diff,
        'up in lactation',up_L_iBAQ,
```

```
        'up in maintenance',up_M_iBAQ)

# membrane prots up in L
T3 <-
  sig_L_iBAQ %>%
  mutate(across(where(is.numeric), ~signif(.x, digits = 2))) %>%
  mutate(description=sub('([^=]) [A-Z][A-Z]=.*','\\1',description)) %>%
  filter(grepl('Mem', Localization))%>%
  dplyr::select(accno, l2FC, description, Localization)



# all prots up in L
T4 <- sig_L_iBAQ %>%
  mutate(across(where(is.numeric), ~signif(.x, digits = 2))) %>%
  mutate(description=sub('([^=]) [A-Z][A-Z]=.*','\\1',description)) %>%
  dplyr::select(accno, l2FC, description, Localization)

T4 %>%
  left_join(uniprot_annotations) %>%
  write_tsv('output/iBAQ_LACT.tsv')

# membrane prots up in M
T5 <- sig_M_iBAQ %>%
  mutate(across(where(is.numeric), ~signif(.x, digits = 2))) %>%
  mutate(description=sub('([^=]) [A-Z][A-Z]=.*','\\1',description)) %>%
  filter(grepl('Mem', Localization)) %>%
  dplyr::select(accno, l2FC, description, Localization)

# All prots up in M
T6 <- sig_M_iBAQ %>%
  mutate(across(where(is.numeric), ~signif(.x, digits = 2))) %>%
  mutate(description=sub('([^=]) [A-Z][A-Z]=.*','\\1',description)) %>%
  dplyr::select(accno, l2FC, description, Localization)
```

```
T6 %>%
  left_join(uniprot_annotations) %>%
  write_tsv('output/iBAQ_MAINT.tsv')



### GO term enrichments ###

########## GO and reference STUFF ###########

# GO_terms = select(GO.db, keys(GO.db, "GOID"), c("TERM", "ONTOLOGY"))







# GO_lact <- GO_all[GO_all$accno %in% lact$accno,]  # These mappings need to contain all proteins not just sigs
# GO_maint <- GO_all[GO_all$accno %in% maint$accno,]

# write_delim(GO_lact, delim = '\t', 'lact_gene2GO.txt')
# write_delim(GO_maint, delim = '\t', 'maint_gene2GO.txt')


uniprot_annotations %>%
  dplyr::select(accno, `Gene Ontology IDs`) %>%
  filter(!is.na(`Gene Ontology IDs`)) %>%
  transmute(accno, GO_ID=gsub('; ', ',', `Gene Ontology IDs`)) %>%
  write_tsv('reference/prot_2_GO.tsv')


Lact_GO_enrich <-
  bind_rows(
```

```
  funfuns::topGO_wrapper(myInterestingGenes = T4$accno, mapping_file = 'reference/prot_2_GO.tsv', ont = 'BP'),
  funfuns::topGO_wrapper(myInterestingGenes = T4$accno, mapping_file = 'reference/prot_2_GO.tsv', ont = 'CC'),
  funfuns::topGO_wrapper(myInterestingGenes = T4$accno, mapping_file = 'reference/prot_2_GO.tsv', ont = 'MF')) %>%
  filter(pval < 0.1) %>%
  dplyr::select(-algorithm, -statistic) %>%
  write_tsv('./output/Lact_GO_enrich.tsv')


Maint_GO_enrich <-
  bind_rows(
    funfuns::topGO_wrapper(myInterestingGenes = T6$accno, mapping_file = 'reference/prot_2_GO.tsv', ont = 'BP'),
    funfuns::topGO_wrapper(myInterestingGenes = T6$accno, mapping_file = 'reference/prot_2_GO.tsv', ont = 'CC'),
    funfuns::topGO_wrapper(myInterestingGenes = T6$accno, mapping_file = 'reference/prot_2_GO.tsv', ont = 'MF')) %>%
  filter(pval < 0.1) %>%
  dplyr::select(-algorithm, -statistic) %>%
  write_tsv('./output/Maint_GO_enrich.tsv')




###


exp_design_tibble <-
  tibble(strain=rep(c('O26', 'O111', 'O145'),4),
         condition=rep(c(rep('vitro', 3), rep('vivo', 3)),2),
         diet=c(rep('Lact', 6), rep('maint', 6)),
         LC_MSMS_run=c(rep('Run1',6), rep('Run2',6)),
         iTRAQ_label=c(1:6, 1:6))
#
```
