# Supplementary material for: Growth dynamics and protein-expression of Escherichia coli serotypes O26:H11, O111:H8 and O145:NM in the bovine rumen
Source: PLoS One. 2025 Jun 4;20(6):e0313978. doi: 10.1371/journal.pone.0313978 (PMC12136435; doi:10.1371/journal.pone.0313978)
Supplement: S2 Text — (PDF) [file pone.0313978.s002.pdf]

Experimental Design:

strain	condition	diet	LC_MSMS_run	iTRAQ_label
O26	vitro	Lact	Run1	1
O111	vitro	Lact	Run1	2
O145	vitro	Lact	Run1	3
O26	vivo	Lact	Run1	4
O111	vivo	Lact	Run1	5
O145	vivo	Lact	Run1	6
O26	vitro	maint	Run2	1
O111	vitro	maint	Run2	2
O145	vitro	maint	Run2	3
O26	vivo	maint	Run2	4
O111	vivo	maint	Run2	5
O145	vivo	maint	Run2	6

```r
library(tidyverse)
library(vegan)
library(broom)
library(NOMAD)


# function for running tests on clr normalized data
run_tests <-
  function(matrix){
    # browser()
    tst <-
      matrix %>%
      as.data.frame() %>%
      rownames_to_column(var='ID') %>%
      separate(ID, into=c('strain', 'condition'),remove = FALSE) %>%
      mutate(condition=factor(condition, levels = c('vitro', 'vivo'))) %>%
      pivot_longer(names_to = 'accno', values_to = 'intensity', -c(ID, strain
, condition)) %>%
      group_by(accno) %>%
      nest() %>%
      mutate(TTEST=map(.x=data,
                       .f=~t.test(formula=intensity ~ condition, data=.x) %>%
tidy())) %>%
      dplyr::select(-data) %>%
      unnest(TTEST) %>%
      ungroup() %>%
      mutate(FDR=p.adjust(p.value, method = 'fdr')) %>%
      arrange(estimate)
    return(tst)
  }


# function for running NOMAD normalization and ttests
NOMAD_diff_expression_tests <-
  function(long_peptides){
```

```
    NOMAD_NORM <- nomadNormalization(y=long_peptides$Abundance, x=long_peptid
es, factors = list('Peptide', 'iTRAQ'))

    nomad_proteins <- nomadAssembleProteins(NOMAD_NORM$y, NOMAD_NORM$x)


    TESTS <-
      nomad_proteins$scores %>%
      as.data.frame() %>%
      rownames_to_column(var='accno') %>%
      pivot_longer(cols = -accno, values_to = 'intensity') %>%
      mutate(reporter=sub('Run1_iTRAQ','',name),
             condition=ifelse(reporter %in% c(1,2,3), 'vitro', 'vivo'),
             condition=factor(condition, levels = c('vitro', 'vivo')),
             strain=case_when(
               reporter %in% c(1,4) ~'O26',
               reporter %in% c(2,5) ~'O111',
               reporter %in% c(3,6) ~'O145'
             )) %>%
      group_by(accno) %>%
      mutate(VAR=var(intensity)) %>%
      filter(VAR != 0) %>%#pull(accno) %>% unique()
      nest() %>%
      mutate(TTEST=map(.x=data,
                       .f=~t.test(formula=intensity ~ condition, data=.x) %>%
tidy())) %>%
      dplyr::select(-data) %>%
      unnest(TTEST) %>%
      ungroup() %>%
      mutate(FDR=p.adjust(p.value, method = 'fdr')) %>%
      arrange(estimate)
    return(TESTS)
  }


run_all_norm_tests <- function(MATRIX, NOMAD_TESTS){
```

```r
MATRIX <- MATRIX[,colSums(MATRIX) != 0]
MATRIX_log <- apply(MATRIX, c(1,2), FUN = log)
MATRIX_log_ratio <- t(scale(t(MATRIX_log), scale=FALSE))
MATRIX_clr <- decostand(x = MATRIX, method = 'clr')
MATRIX_rab <- decostand(x = MATRIX, method = 'total')
MATRIX_log_rab <- decostand(x = MATRIX_log, method = 'total')

# hist(MATRIX_log_rab)

raw_tests <-
  run_tests(MATRIX) %>%
  mutate(order=1:n(),
         method='raw')



nomad_tests <-
  NOMAD_TESTS %>%
  mutate(order=1:n(),
         method='nomad')

clr_tests <-
  run_tests(MATRIX_clr) %>%
  mutate(order=1:n(),
         method='clr')

rab_tests <-  run_tests(MATRIX_rab) %>%
  mutate(order=1:n(),
         method='rab')

log_rab_tests <-  run_tests(MATRIX_log_rab) %>%
  mutate(order=1:n(),
         method='log_rab')

log_tests <-
```

```
    run_tests(MATRIX_log) %>%
    mutate(order=1:n(),
           method='log')




  all_tests <-
    bind_rows(raw_tests, nomad_tests,clr_tests, rab_tests, log_rab_tests, log
_tests) %>%
    group_by(accno) %>%
    mutate(MEAN_ORDER=mean(order),
           ref_order=order[method=='clr']) %>%
    mutate(SIG=ifelse(p.value < 0.05, T, F))

  return(all_tests)


}



run_multivariate <-
  function(MATRIX){
    META <-
      tibble(ID=rownames(MATRIX)) %>%
      separate(ID, into=c('strain', 'condition'), remove = F)



    plot(metaMDS(MATRIX, distance = 'robust.aitchison', autotransform = FALSE
))
    NMDS <- metaMDS(MATRIX, distance = 'robust.aitchison', autotransform = FA
LSE)

    prot_NMDS_coords <- NMDS$species %>% as.data.frame() %>% rownames_to_colu
mn(var='accno')
```

```
    META <-
      NMDS$points %>%
      as.data.frame() %>%
      rownames_to_column(var='ID') %>%
      left_join(META)

    NMDS_plot <-
      META %>%
      ggplot(aes(x=MDS1, y=MDS2, fill=condition, label=strain)) +
      geom_point(size=3, shape=21)+
      geom_label_repel(force = 10,box.padding = unit(1,'lines'), min.segment.
length = unit(0, 'lines')) +
      theme_bw() +
      lims(x=c(-8,8), y=c(-6,6)) +
      ggtitle('NMDS of Aitchison distances')
    NMDS_plot

    adonis_results <- adonis2(method = 'robust.aitchison',data = META, formul
a = iTRAQ_L_mat ~ strain * condition)
    return(list(NMDS_plot, adonis_results))
  }



### strainwise comparisons ###



strain_comparisons <- function(MATRIX){
  # browser()

  MATRIX <- MATRIX[,colSums(MATRIX) != 0]
  MATRIX_clr <- decostand(x = MATRIX, method = 'clr')

  RESULTS <-
    MATRIX_clr %>%
```

```
    as.data.frame() %>%
    rownames_to_column(var='ID') %>%
    pivot_longer(cols=-ID, names_to = 'accno', values_to = 'clr') %>%
    mutate(strain=sub('(.*)_(.*)','\\1',ID),
           condition=sub('(.*)_(.*)','\\2',ID)) %>%
    group_by(strain, accno) %>%
    summarise(condition_dif=clr[2] - clr[1],
              enriched_in=ifelse(condition_dif > 0 , condition[2], condition[
1]),
              .groups='drop') #%>%
    # pull(strain_ratio) %>% hist()
  return(RESULTS)



}



# annotations from uniprot


uniprot_annotations <- read_tsv('output/pan_0.05_annotations.tsv') %>%
  mutate(accno=From) %>%
  dplyr::select(accno, everything(), -From, -`pH dependence`)

prot_descipts <- read_tsv('output/protein_descriptions.tsv')

# output of maxquant, protein groups
# filtered to only consider proteins that make the cutoffs


PG <-
  read_tsv('output/protein_groups_cleaned.tsv') %>%
  filter(`Q-value` < 0.05) %>%
  filter(Peptides > 1) %>%
  filter(!grepl('CON__', accno))

valid_accnos <- PG %>% pull(accno)
```

```
##########


# Peptide level intensities for NOMAD

#

# ALL PEPTIDES <- read tsv('maxquant results/combined/txt/peptides.txt')

ALL PEPTIDES <- read tsv('maxquant results pan pro 0.05 pep 0.01/combined/txt
/peptides.txt')


ALL_PEPTIDES <-

  ALL_PEPTIDES %>%

  dplyr::select(Sequence,`Leading razor protein`,  matches('Reporter intensit
y . [a-z]+')) %>%

  mutate(Protein=`Leading razor protein`,

         Peptide=Sequence) %>%

  dplyr::select(Protein, Peptide,everything(), -`Leading razor protein`, -Seq
uence) %>%

  mutate(

    tmp_id=str_split(gsub('REV__tr','',Protein), pattern = '\\|'),

    first_underscore=map_int(tmp_id, ~min(grep('_', .x)))) %>%

  # dplyr::select(first underscore, tmp id) %>%

  mutate(TMP=map2_chr(tmp_id,first_underscore, ~pluck(.x,.y) ),

         TMP=sub(';$','',TMP),

         TMP=sub(';[trsp]+$','',TMP),

         TMP=sub('REV__', '', TMP),

         Protein=TMP) %>%

  dplyr::select(-first_underscore, -tmp_id, -TMP) %>%

  filter(Protein %in% valid_accnos)




LONG_PEPTIDES <-

  ALL_PEPTIDES %>%

  pivot_longer(cols = -c(Protein, Peptide), names_to = 'Sample', values_to =
'Abundance') %>%

  mutate(iTRAQ=sub('Reporter intensity ([0-8]+) [a-z]+','\\1',Sample),

         Run=sub('Reporter intensity ([0-8]+) ([a-z]+)','\\2',Sample),) %>%
```

```r
  dplyr::select(-Sample) %>%
  filter(iTRAQ %in% c(1:6))%>%
  filter(!grepl('CON', Protein))
# summary stats


protein_summary <-
  LONG_PEPTIDES %>%
  filter(Abundance > 0) %>%
  group_by(Run,Protein) %>%
  summarise(num peptides=sum(length(unique(Peptide))), .groups = 'drop')

run_summary <-
  protein_summary %>%
  filter(num_peptides >1) %>%
  group_by(Run) %>%
  summarise(tot_proteins=sum(length(unique(Protein))))



unique_proteins <-
  protein_summary %>%
  filter(num_peptides >1) %>%
  summarise(lactation=sum(!(Protein[Run == 'lactation'] %in% Protein[Run == '
maintenance'])),
            maintenance=sum(!(Protein[Run == 'maintenance'] %in% Protein[Run
== 'lactation'])),
            both=sum((Protein[Run == 'lactation'] %in% Protein[Run == 'mainte
nance']))) %>%
  pivot_longer(cols=everything(),names_to = 'Run', values_to = 'unique_protei
ns')

### USE THIS TABLE
unique_proteins %>% add_row(Run='total', unique_proteins=sum(unique_proteins$
unique_proteins))
```

```
## # A tibble: 4 × 2
```

```
##    Run          unique_proteins
##    <chr>                  <int>
## 1 lactation                 70
## 2 maintenance               70
## 3 both                     243
## 4 total                    383
```

```
lact_peptides <-
  LONG_PEPTIDES %>%
  filter(Run == 'lactation' & Abundance > 0) %>%
  # filter(Run == 'lactation') %>%
  mutate(Run=1)


maint_peptides <-
  LONG_PEPTIDES %>%
  filter(Run == 'maintenance'& Abundance > 0) %>%
  # filter(Run == 'lactation') %>%
  mutate(Run=1)




NOMAD LACT TESTS <- NOMAD diff expression tests(long peptides = lact peptides
)
```

```
## Running normalization with  9186  number of data points
## Normalizing for factor:  Peptide
## Normalizing for factor:  iTRAQ
```

```
NOMAD_MAINT_TESTS <- NOMAD_diff_expression_tests(long_peptides = maint_peptid
es)
```

```
## Running normalization with  8658  number of data points
## Normalizing for factor:  Peptide
## Normalizing for factor:  iTRAQ
```

```
p_NOMAD_LACT_HIST <-
  NOMAD_LACT_TESTS %>%
  # filter(p.value < 0.05) %>%
  ggplot(aes(x=estimate, fill=ifelse(p.value < 0.05, T, F))) +
```

```
  annotate(x=2, y=50, geom='label', label='Enriched in vivo')+
  annotate(x=-2, y=50, geom='label', label='Enriched in vitro')+
  geom_histogram() + xlim(-3,3)+
  geom_vline(xintercept = 0) +
  labs(y='count',
       fill='P < 0.05')+
  theme(legend.position = 'bottom')

p_NOMAD_MAINT_HIST <-
  NOMAD_MAINT_TESTS %>%
  # filter(p.value < 0.05) %>%
  ggplot(aes(x=estimate, fill=ifelse(p.value < 0.05, T, F))) +
  annotate(x=2, y=50, geom='label', label='Enriched in vivo')+
  annotate(x=-2, y=50, geom='label', label='Enriched in vitro')+
  geom_histogram() + xlim(-3,3)+
  geom_vline(xintercept = 0) +
  labs(y='count',
       fill='P < 0.05')+
  theme(legend.position = 'bottom')
# All 'sigs' are more abundant in the vivo condition in both the lact and mai
nt diets




# This section for t.tests with other normalization techniques
#
# tibble(strain=paste0('Strain', rep(c(1:3),4)),
#         condition=rep(c(rep('vivo', 3), rep('vitro', 3)),2),
#         diet=c(rep('Lact', 6), rep('maint', 6)),
#         LC_MSMS_run=c(rep('Run1',6), rep('Run2',6)),
#         iTRAQ_label=c(1:6, 1:6))
#

iTRAQ <-
  PG %>%
```

```
  dplyr::select(accno, contains('Reporter intensity corrected')) %>%
  pivot_longer(-accno, names_to = c('reporter', 'treatment'),
               names_prefix = 'Reporter intensity corrected ',
               names_sep = ' ', values_to = 'intensity')

#  itraq reporter intensities
iTRAQ %>%
  ggplot(aes(x=reporter, y=intensity)) +
  geom_col(color='black') +
  facet_wrap(~treatment)
```

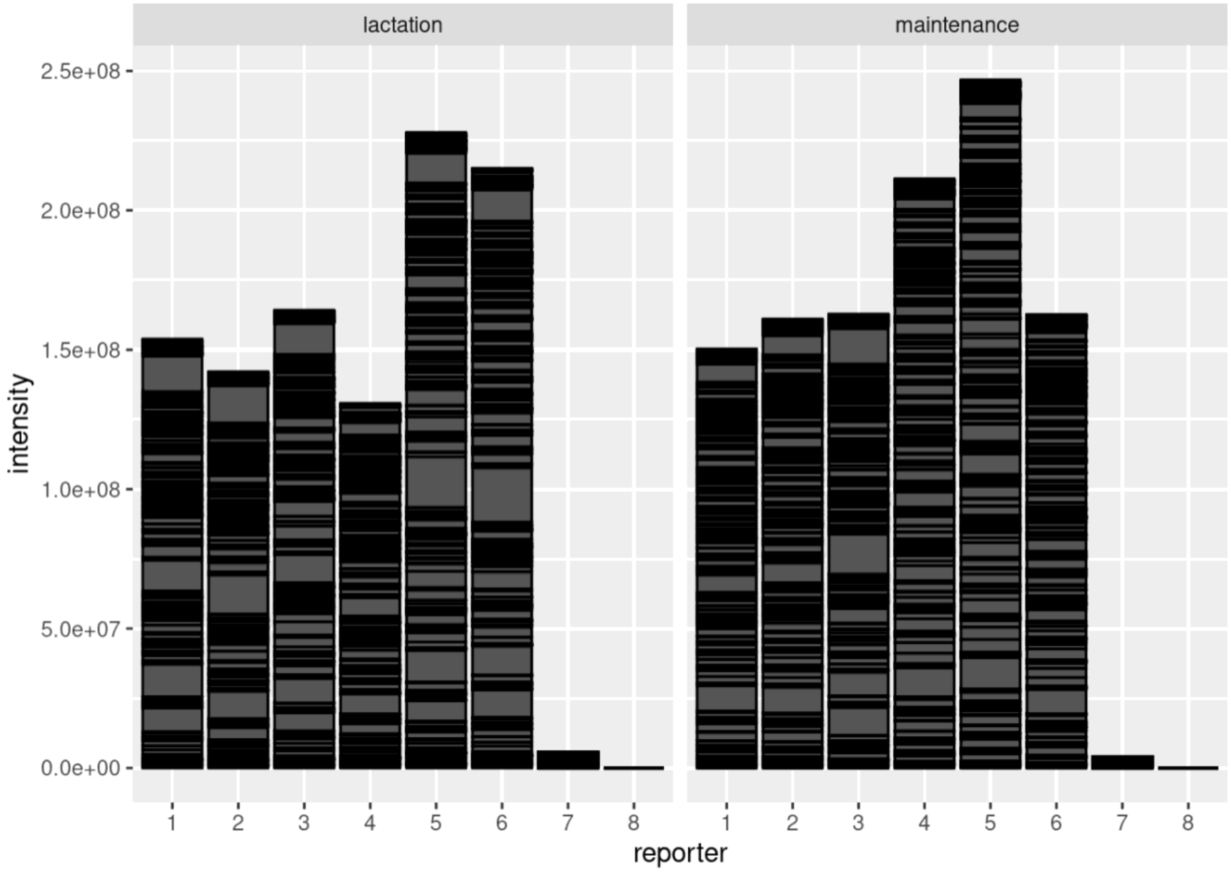


```
iTRAQ <-
  iTRAQ %>%
  filter(reporter %in% c(1:6)) %>% # only 6 of the 8 reporters used
  group_by(reporter, treatment) %>%
  mutate(rel_intensity=intensity/sum(intensity),
```

```
        log intensity=log(intensity),
        # log_ratio_intensity=log_intensity/sum(log_intensity),
        condition=ifelse(reporter %in% c(1,2,3), 'vitro', 'vivo'),
        strain=case_when(
          reporter %in% c(1,4) ~'O26',
          reporter %in% c(2,5) ~'O111',
          reporter %in% c(3,6) ~'O145'
        )) %>%
  ungroup()

# this shows us that within each MSMS run we only dectect proteins that
# were detectable in all strains across both in-vivo and in-vitro conditions.

# iTRAQ %>%
#   group_by(treatment, accno) %>%
#   summarise(ANY=any(intensity == 0),
#             ALL=all(intensity == 0)) %>%
#   ungroup() %>%
#   mutate(ANY_NOT_ALL=ANY & !ALL) %>%
#   filter(ANY_NOT_ALL)

###

iTRAQ_L <-
  iTRAQ %>%
  filter(treatment == 'lactation' & is.finite(log_intensity)) %>%
  group_by(reporter) %>%
  mutate(norm_log_intensity=log_intensity - mean(log_intensity)) %>%
  ungroup()



iTRAQ_L %>%
  ggplot(aes(x=reporter, y=norm_log_intensity, fill=condition)) +
```

```
  geom col(color='black') +
  facet_wrap(~treatment)
```

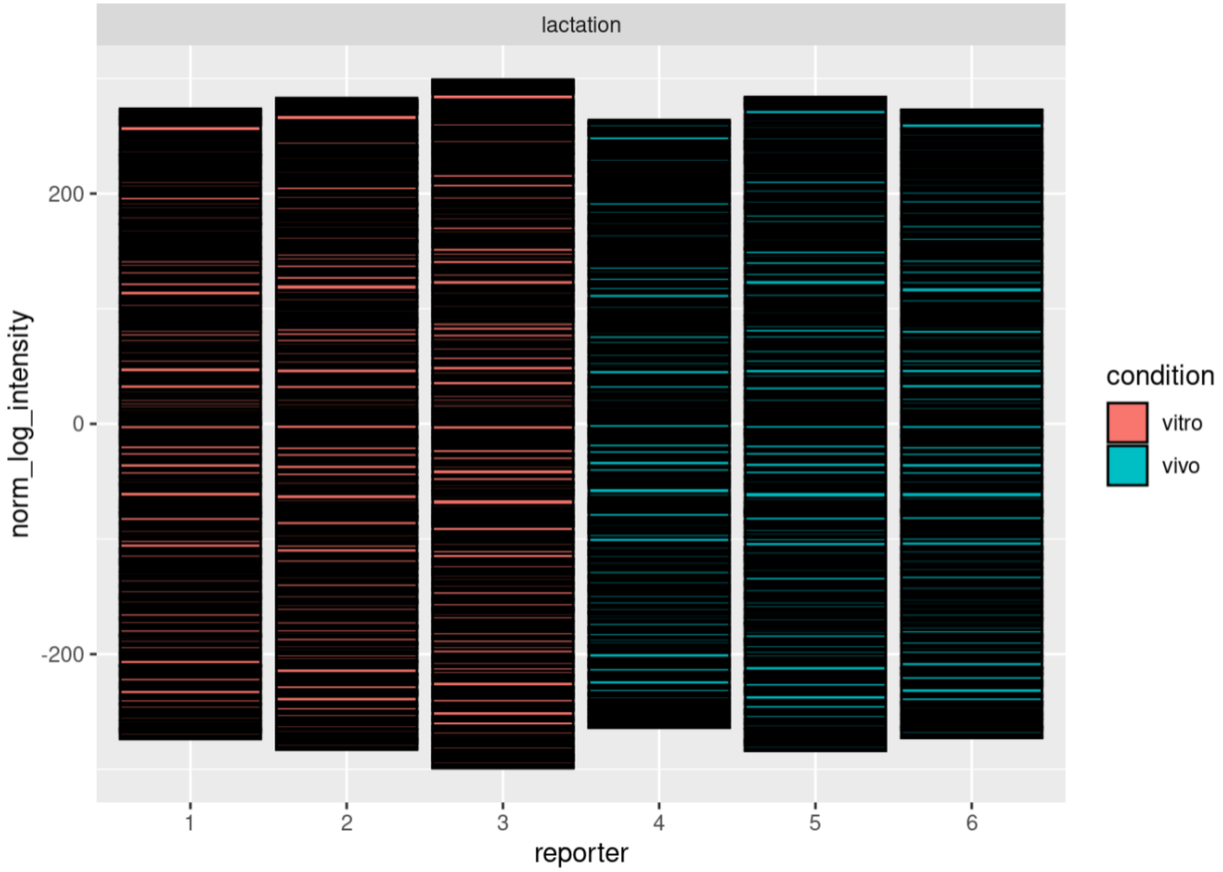


```
iTRAQ_L_mat <-
  iTRAQ_L %>%
  dplyr::select(accno, strain, condition, intensity) %>%
  pivot wider(names from = c(strain, condition), values from = intensity ) %>
%
   column_to_rownames('accno') %>% as.matrix() %>% t()

iTRAQ_L_mat <- iTRAQ_L_mat[,colSums(iTRAQ_L_mat) != 0]

 iTRAQ_M <- iTRAQ %>% filter(treatment == 'maintenance')


iTRAQ_M_mat <-
  iTRAQ_M %>%
```

```
  dplyr::select(accno, strain, condition, intensity) %>%
  pivot_wider(names_from = c(strain, condition), values_from = intensity ) %>
%
  column_to_rownames('accno') %>% as.matrix() %>% t()


iTRAQ_M_mat <- iTRAQ_M_mat[,colSums(iTRAQ_M_mat) != 0]




# Run all normalizations and tests:
# need to supply nomad tests separately



lact all tests <- run all norm tests(MATRIX =iTRAQ L mat, NOMAD TESTS = NOMAD
 LACT TESTS )
lact_all_tests <- lact_all_tests %>% left_join(prot_descipts)


lact_all_tests %>% filter(method == 'clr') %>% filter(FDR < 0.05)
## # A tibble: 0 × 18
## # Groups:   accno [0]
## # … with 18 variables: accno <chr>, estimate <dbl>, estimate1 <dbl>,
## #   estimate2 <dbl>, statistic <dbl>, p.value <dbl>, parameter <dbl>,
## #   conf.low <dbl>, conf.high <dbl>, method <chr>, alternative <chr>,
## #   FDR <dbl>, order <int>, MEAN_ORDER <dbl>, ref_order <int>, SIG <lgl>,
## #   description <chr>, Localization <chr>
lact_all_tests %>% filter(method == 'nomad') %>% filter(FDR < 0.05)
## # A tibble: 0 × 18
## # Groups:   accno [0]
## # … with 18 variables: accno <chr>, estimate <dbl>, estimate1 <dbl>,
## #   estimate2 <dbl>, statistic <dbl>, p.value <dbl>, parameter <dbl>,
## #   conf.low <dbl>, conf.high <dbl>, method <chr>, alternative <chr>,
## #   FDR <dbl>, order <int>, MEAN_ORDER <dbl>, ref_order <int>, SIG <lgl>,
## #   description <chr>, Localization <chr>
p lact tests <-
  lact_all_tests %>%
```

```r
  filter(method %in% c('clr', 'nomad')) %>%
  ggplot(aes(x=estimate, fill=SIG))+
  geom_histogram()+
  facet_wrap(~method, scales = 'free') +
  geom_vline(xintercept = 0) +
  labs(fill='uncorrected P < 0.05') +
  theme(legend.position = 'bottom') +
  ggtitle('Histograms of log2(fold change) for all proteins',
          'Negative estimates indicate greater expression in-vitro')


lact_all_tests <-
  lact_all_tests%>%
  ungroup() %>%
  transmute(gene_name = accno,
            method,
            estimate,
            p.value,
            FDR,description, Localization ) %>%
  mutate(across(where(is.numeric), ~signif(.x, digits = 2))) %>%
  mutate(description=sub('([^=]) [A-Z][A-Z]=.*','\\1',description))




CLR_LACT_VIVO <-
  lact_all_tests %>%
  filter(method == 'clr') %>%
  filter(p.value < 0.05) %>%
  arrange(FDR) %>%
  filter(estimate > 0)

CLR_LACT_VIVO %>%
  left_join(uniprot_annotations, by = c('gene_name' = 'accno')) %>%
```

```
  write_tsv('output/CLR_LACT_VIVO.tsv')


CLR_LACT_VITRO <-
  lact_all_tests %>%
  filter(method == 'clr') %>%
  filter(p.value < 0.05) %>%
  arrange(FDR) %>%
  filter(estimate < 0)

CLR_LACT_VITRO %>%
  left_join(uniprot_annotations, by = c('gene_name' = 'accno')) %>%
  write_tsv('output/CLR_LACT_VITRO.tsv')

## for lact venn table

# num up in vivo
LACT_NUM_UP_VIVO <- nrow(CLR_LACT_VIVO)

# num up in vitro
LACT_NUM_UP_VITRO <- nrow(CLR_LACT_VITRO)

# num not different
LACT_NOT_DIFF <- ncol(iTRAQ_L_mat) - (LACT_NUM_UP_VITRO + LACT_NUM_UP_VIVO)
# 343

# if using FDR corrected pvalues
# all 405 proteins not different
ncol(iTRAQ_L_mat)
```

```
## [1] 405
```

```
uncorrectedPv_lact_itraq_table <-
  tibble(enriched_in=c('vivo', 'vitro', 'not different'),
         num_proteins=c(LACT_NUM_UP_VIVO, LACT_NUM_UP_VITRO, LACT_NOT_DIFF))
```

```
correctedPv_lact_itraq_table <-
  tibble(enriched_in=c('vivo', 'vitro', 'not different'),
         num proteins=c(0, 0, ncol(iTRAQ L mat)))


###


NOMAD_LACT_VIVO <-
  lact_all_tests %>%
  filter(method == 'nomad') %>%
  filter(p.value < 0.05) %>%
  arrange(FDR) %>%
  filter(estimate > 0)

NOMAD_LACT_VIVO %>%
  left_join(uniprot_annotations, by = c('gene_name' = 'accno')) %>%
  write_tsv('output/NOMAD_LACT_VIVO.tsv')


NOMAD_LACT_VITRO <-
  lact_all_tests %>%
  filter(method == 'nomad') %>%
  filter(p.value < 0.05) %>%
  arrange(FDR) %>%
  filter(estimate < 0)

NOMAD_LACT_VITRO %>%
  left_join(uniprot_annotations, by = c('gene_name' = 'accno')) %>%
  write_tsv('output/NOMAD_LACT_VITRO.tsv')
```

```
maint_all_tests <- run_all_norm_tests(MATRIX =iTRAQ_M_mat, NOMAD_TESTS = NOMA
D_MAINT_TESTS )

maint_all_tests <- maint_all_tests %>% left_join(prot_descipts)


# only one sig in maint (nomad)

maint_all_tests %>% filter(method == 'clr') %>% filter(FDR < 0.05)
```

```
## # A tibble: 0 × 18
## # Groups:   accno [0]
## # … with 18 variables: accno <chr>, estimate <dbl>, estimate1 <dbl>,
## #   estimate2 <dbl>, statistic <dbl>, p.value <dbl>, parameter <dbl>,
## #   conf.low <dbl>, conf.high <dbl>, method <chr>, alternative <chr>,
## #   FDR <dbl>, order <int>, MEAN_ORDER <dbl>, ref_order <int>, SIG <lgl>,
## #   description <chr>, Localization <chr>
```

```
maint_all_tests %>% filter(method == 'nomad') %>% filter(FDR < 0.05)
```

```
## # A tibble: 1 × 18
## # Groups:   accno [1]
##   accno    estim…¹ estim…² estim…³ stati…⁴ p.value param…⁵ conf.…⁶ conf.…⁷ me
thod
##   <chr>      <dbl>   <dbl>   <dbl>   <dbl>   <dbl>   <dbl>   <dbl>   <dbl>
<chr>
## 1 W1W747…  -0.773 -0.0590   0.714   -32.2 4.45e-5    3.15  -0.848  -0.699
nomad
## # … with 8 more variables: alternative <chr>, FDR <dbl>, order <int>,
## #   MEAN_ORDER <dbl>, ref_order <int>, SIG <lgl>, description <chr>,
## #   Localization <chr>, and abbreviated variable names ¹estimate, ²estimat
e1,
## #   ³estimate2, ⁴statistic, ⁵parameter, ⁶conf.low, ⁷conf.high
```

```
p_maint_tests <-
  maint_all_tests %>%
  filter(method %in% c('clr', 'nomad')) %>%
  ggplot(aes(x=estimate, fill=SIG))+
  geom_histogram()+
  facet_wrap(~method, scales = 'free') +
  geom_vline(xintercept = 0) +
  labs(fill='uncorrected P < 0.05') +
```

```
  theme(legend.position = 'bottom') +
  ggtitle('Histograms of log2(fold change) for all proteins',
          'Negative estimates indicate greater expression in-vitro')




maint_all_tests <-
  maint_all_tests %>% ungroup() %>%
  transmute(gene_name = accno,
            method,
            estimate,
            p.value,
            FDR,description, Localization ) %>%
  mutate(across(where(is.numeric), ~signif(.x, digits = 2))) %>%
  mutate(description=sub('([^=]) [A-Z][A-Z]=.*','\\1',description))

CLR_MAINT_VIVO <-
  maint_all_tests %>%
  filter(method == 'clr') %>%
  filter(p.value < 0.05) %>%
  arrange(FDR) %>%
  filter(estimate > 0)

CLR_MAINT_VIVO %>%
  left_join(uniprot_annotations, by = c('gene_name' = 'accno')) %>%
  write_tsv('output/CLR_MAINT_VIVO.tsv')



CLR_MAINT_VITRO <-
  maint_all_tests %>%
  filter(method == 'clr') %>%
  filter(p.value < 0.05) %>%
  arrange(FDR) %>%
```

```
  filter(estimate < 0)


CLR_MAINT_VITRO %>%
  left_join(uniprot_annotations, by = c('gene_name' = 'accno')) %>%
  write_tsv('output/CLR_MAINT_VITRO.tsv')


## for maint venn table


nrow(CLR_MAINT_VIVO)
## [1] 26
nrow(CLR_MAINT_VITRO)
## [1] 41
## for lact venn table


# num up in vivo
MAINT_NUM_UP_VIVO <- nrow(CLR_MAINT_VIVO)


# num up in vitro
MAINT_NUM_UP_VITRO <- nrow(CLR_MAINT_VITRO)


# num not different
MAINT NOT DIFF <- ncol(iTRAQ M mat) - (MAINT NUM UP VITRO + MAINT NUM UP VIVO
)
#


# if using FDR corrected pvalues
# all 412 proteins not different
ncol(iTRAQ_M_mat)
## [1] 412
uncorrectedPv_maint_itraq_table <-
  tibble(enriched_in=c('vivo', 'vitro', 'not different'),
         num_proteins=c(MAINT_NUM_UP_VIVO, MAINT_NUM_UP_VITRO, MAINT_NOT_DIFF
))
```

```
correctedPv_maint_itraq_table <-
  tibble(enriched_in=c('vivo', 'vitro', 'not different'),
         num_proteins=c(0, 0, ncol(iTRAQ_M_mat)))


###

NOMAD_MAINT_VIVO <-
  maint_all_tests %>%
  filter(method == 'nomad') %>%
  filter(p.value < 0.05) %>%
  arrange(FDR) %>%
  filter(estimate > 0)

NOMAD_MAINT_VIVO %>%
  left_join(uniprot_annotations, by = c('gene_name' = 'accno')) %>%
  write_tsv('output/NOMAD_MAINT_VIVO.tsv')


NOMAD_MAINT_VITRO <-
  maint_all_tests %>%
  filter(method == 'nomad') %>%
  filter(p.value < 0.05) %>%
  arrange(FDR) %>%
  filter(estimate < 0)

NOMAD_MAINT_VIVO %>%
  left_join(uniprot_annotations, by = c('gene_name' = 'accno')) %>%
  write_tsv('output/NOMAD_MAINT_VITRO.tsv')
```

```
# pull out nomad and clr data for each




### end differential abundance

# multivariate similarity

lact_multivariate <- run_multivariate(MATRIX =iTRAQ_L_mat)
```

```
## Run 0 stress 0.06950403
## Run 1 stress 0.09888119
## Run 2 stress 0.09888119
## Run 3 stress 0.09888119
## Run 4 stress 8.916812e-05
## ... New best solution
## ... Procrustes: rmse 0.2514416  max resid 0.3972699
## Run 5 stress 9.733161e-05
## ... Procrustes: rmse 4.962573e-05  max resid 8.425069e-05
## ... Similar to previous best
## Run 6 stress 0.1511089
## Run 7 stress 0.1516376
## Run 8 stress 0.1574259
## Run 9 stress 8.727043e-05
## ... New best solution
## ... Procrustes: rmse 4.002447e-05  max resid 6.819927e-05
## ... Similar to previous best
## Run 10 stress 0.2340151
## Run 11 stress 0.09888119
## Run 12 stress 0.1547916
## Run 13 stress 0.1511089
## Run 14 stress 8.846009e-05
## ... Procrustes: rmse 1.790918e-05  max resid 3.201566e-05
## ... Similar to previous best
```

```
## Run 15 stress 9.733246e-05
## ... Procrustes: rmse 9.518298e-05  max resid 0.0001102307
## ... Similar to previous best
## Run 16 stress 0.06950403
## Run 17 stress 0.09888119
## Run 18 stress 9.064786e-05
## ... Procrustes: rmse 8.585204e-05  max resid 0.0001047341
## ... Similar to previous best
## Run 19 stress 0.1547916
## Run 20 stress 0.2761341
## *** Best solution repeated 4 times
```

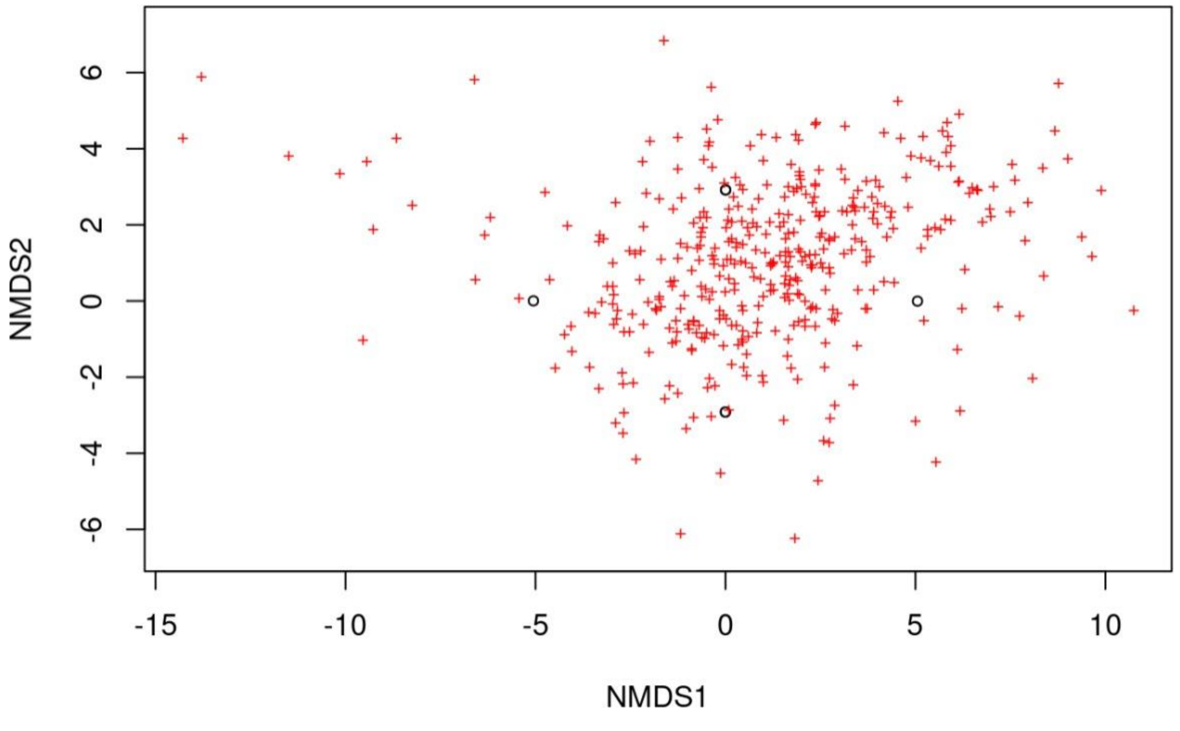


```
## Run 0 stress 0.06950403
## Run 1 stress 0.06950403
## ... New best solution
## ... Procrustes: rmse 7.220788e-07  max resid 9.432625e-07
```

```
## ... Similar to previous best
## Run 2 stress 0.2181459
## Run 3 stress 0.06950403
## ... Procrustes: rmse 9.992115e-07  max resid 1.494649e-06
## ... Similar to previous best
## Run 4 stress 9.987689e-05
## ... New best solution
## ... Procrustes: rmse 0.2514362  max resid 0.3972298
## Run 5 stress 0.2673072
## Run 6 stress 0.1574259
## Run 7 stress 0.09888119
## Run 8 stress 0.2673072
## Run 9 stress 9.598933e-05
## ... New best solution
## ... Procrustes: rmse 2.36592e-05  max resid 4.023265e-05
## ... Similar to previous best
## Run 10 stress 0.09888119
## Run 11 stress 0.1431705
## Run 12 stress 0.09888119
## Run 13 stress 0.09888119
## Run 14 stress 7.613847e-05
## ... New best solution
## ... Procrustes: rmse 6.130332e-05  max resid 0.0001145663
## ... Similar to previous best
## Run 15 stress 0.09888119
## Run 16 stress 9.274181e-05
## ... Procrustes: rmse 4.946741e-05  max resid 9.697384e-05
## ... Similar to previous best
## Run 17 stress 8.618249e-05
## ... Procrustes: rmse 6.77033e-05  max resid 0.0001020608
## ... Similar to previous best
## Run 18 stress 8.052902e-05
## ... Procrustes: rmse 1.248378e-05  max resid 2.478298e-05
## ... Similar to previous best
```

```
## Run 19 stress 0.06950403
## Run 20 stress 9.029333e-05
## ... Procrustes: rmse 4.610653e-05  max resid 8.920084e-05
## ... Similar to previous best
## *** Best solution repeated 5 times
```

```
maint_multivariate <- run_multivariate(MATRIX = iTRAQ_M_mat)
```

```
## Run 0 stress 0
## Run 1 stress 4.175602e-05
## ... Procrustes: rmse 0.1395792  max resid 0.2282503
## Run 2 stress 2.571119e-05
## ... Procrustes: rmse 0.09986866  max resid 0.1743476
## Run 3 stress 9.556024e-05
## ... Procrustes: rmse 0.0619926  max resid 0.08558483
## Run 4 stress 0.1466932
## Run 5 stress 9.150028e-05
## ... Procrustes: rmse 0.1235718  max resid 0.1799293
## Run 6 stress 9.497333e-05
## ... Procrustes: rmse 0.05307149  max resid 0.08889346
## Run 7 stress 0
## ... Procrustes: rmse 0.07851493  max resid 0.1313054
## Run 8 stress 0
## ... Procrustes: rmse 0.05865634  max resid 0.09171743
## Run 9 stress 0.1020843
## Run 10 stress 7.032089e-06
## ... Procrustes: rmse 0.1039162  max resid 0.1581109
## Run 11 stress 0.1466932
## Run 12 stress 0
## ... Procrustes: rmse 0.04300038  max resid 0.08466444
## Run 13 stress 9.31757e-05
## ... Procrustes: rmse 0.06045705  max resid 0.08846478
## Run 14 stress 2.368803e-05
## ... Procrustes: rmse 0.05910936  max resid 0.103739
## Run 15 stress 0.1020843
## Run 16 stress 8.139332e-05
```

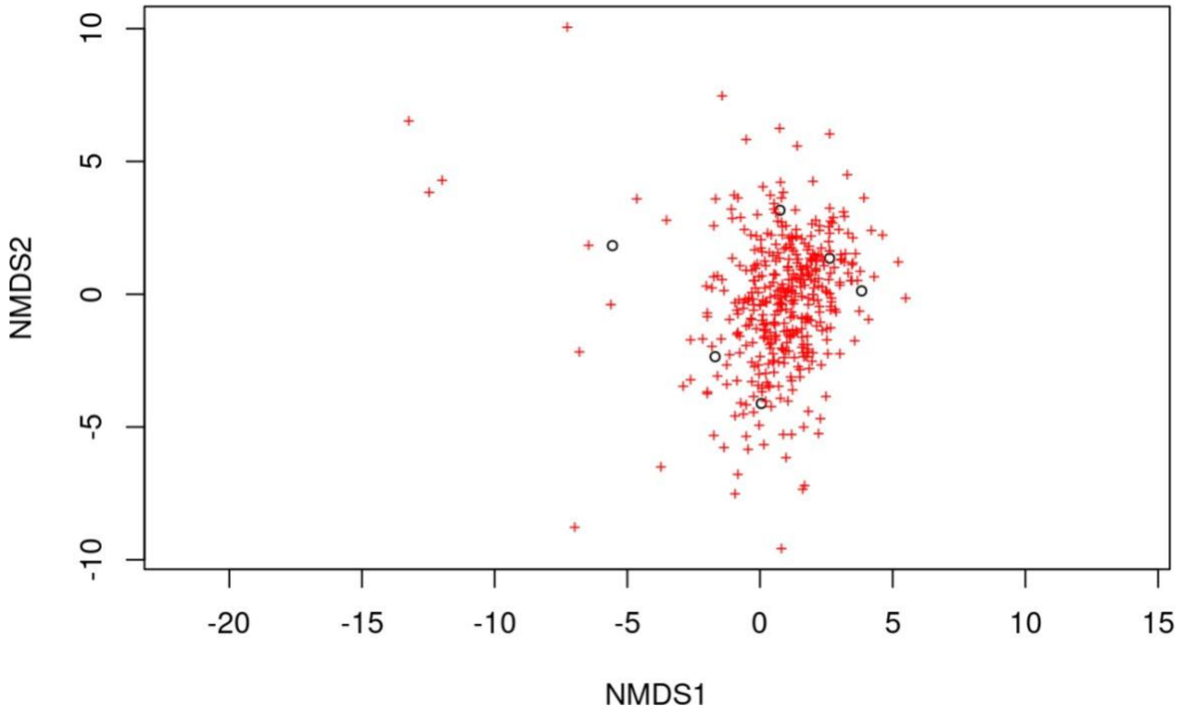

10
5
0
-5
-10
NMDS2
-20
-15
-10
-5
0
5
10
15
NMDS1

```
## Run 0 stress 0
## Run 1 stress 0.2181459
## Run 2 stress 4.141315e-05
## ... Procrustes: rmse 0.1232055  max resid 0.1960731
## Run 3 stress 7.419384e-05
## ... Procrustes: rmse 0.1203489  max resid 0.1840873
## Run 4 stress 0.2761342
## Run 5 stress 3.420277e-05
## ... Procrustes: rmse 0.1604028  max resid 0.2438271
## Run 6 stress 0
## ... Procrustes: rmse 0.05736236  max resid 0.09047772
## Run 7 stress 6.964532e-05
## ... Procrustes: rmse 0.1164636  max resid 0.1787589
## Run 8 stress 0
## ... Procrustes: rmse 0.1045287  max resid 0.1614703
## Run 9 stress 9.451043e-05
## ... Procrustes: rmse 0.1647028  max resid 0.2492411
## Run 10 stress 8.656891e-05
## ... Procrustes: rmse 0.1297161  max resid 0.2239737
## Run 11 stress 3.895858e-05
## ... Procrustes: rmse 0.03662093  max resid 0.05128895
## Run 12 stress 2.067912e-05
## ... Procrustes: rmse 0.07760856  max resid 0.1151195
## Run 13 stress 0.1020843
## Run 14 stress 0.2181459
## Run 15 stress 0
## ... Procrustes: rmse 0.05459718  max resid 0.08135204
## Run 16 stress 7.921436e-06
## ... Procrustes: rmse 0.08909557  max resid 0.1525254
## Run 17 stress 9.405322e-05
## ... Procrustes: rmse 0.1496273  max resid 0.2253367
## Run 18 stress 0.1020843
## Run 19 stress 6.790864e-05
## ... Procrustes: rmse 0.1359032  max resid 0.2049361
```

```
## Run 20 stress 6.615056e-05
## ... Procrustes: rmse 0.08766581  max resid 0.1460576
## *** Best solution was not repeated -- monoMDS stopping criteria:
##     15: stress < smin
##      2: stress ratio > sratmax
##      3: scale factor of the gradient < sfgrmin
```

```
### strain ratio analysis

maint_within_strain_comps <-
  strain_comparisons(MATRIX = iTRAQ_M_mat)




maint_prot_SDs <-
  maint_within_strain_comps %>%
  dplyr::select(-enriched_in) %>% group_by(accno) %>%
  summarise(SD=sd(condition_dif)) %>%
  arrange(desc(SD))

### NEED TO RE-ORG THIS AND JOIN IN ANNOTATIONS FOR OUTPUT TABLE
library(ComplexHeatmap)

p_maint_within_strain <-
  maint_within_strain_comps %>%
  pivot_wider(-enriched_in, names_from = strain, values_from = condition_dif)
%>%
  left_join(maint_prot_SDs) %>%
  arrange(desc(SD)) %>%
  left_join(uniprot_annotations) %>%
  write_tsv('output/maint_within_strains.tsv') %>%
  slice_max(SD, n=50) %>%
  dplyr::select(accno, O111, O145,O26 ) %>%
  column_to_rownames(var='accno') %>%
  as.matrix() %>%# hist()
  ComplexHeatmap::Heatmap(name = 'Log2(Vivo / Vitro)',
```

```
                          column title = 'Maintenance: within strain ratios b
etween conditions.
                          top 50 proteins with most variation between strains
,
                          (proteins where strains behave differently between
conditions)',
                          row_names_gp = gpar(fontsize = 8))



# pivot_wider(names_from = 'strain', values_from = 'condition_dif')



lact_within_strain_comps <-
  strain_comparisons(MATRIX = iTRAQ_L_mat)


p_lact_within_strain <-
  lact_within_strain_comps %>%
  pivot wider(-enriched in, names from = strain, values from = condition dif)
%>%
  left_join(maint_prot_SDs) %>%
  arrange(desc(SD)) %>%
  left_join(uniprot_annotations) %>%
  write_tsv('output/lact_within_strains.tsv') %>%
  slice_max(SD, n=50) %>%
  dplyr::select(accno, O111, O145,O26 ) %>%
  column_to_rownames(var='accno') %>%
  as.matrix() %>%# hist()
  ComplexHeatmap::Heatmap(name = 'Log2(Vivo / Vitro)',
                          column title = 'Lactation: within strain ratios bet
ween conditions.
                          top 50 proteins with most variation between strains
,
                          (proteins where strains behave differently between
conditions)',
                          row_names_gp = gpar(fontsize = 8))
```
