# Supplementary figures and images for: Growth dynamics and protein-expression of Escherichia coli serotypes O26:H11, O111:H8 and O145:NM in the bovine rumen
Source: PLoS One. 2025 Jun 4;20(6):e0313978. doi: 10.1371/journal.pone.0313978 (PMC12136435; doi:10.1371/journal.pone.0313978)

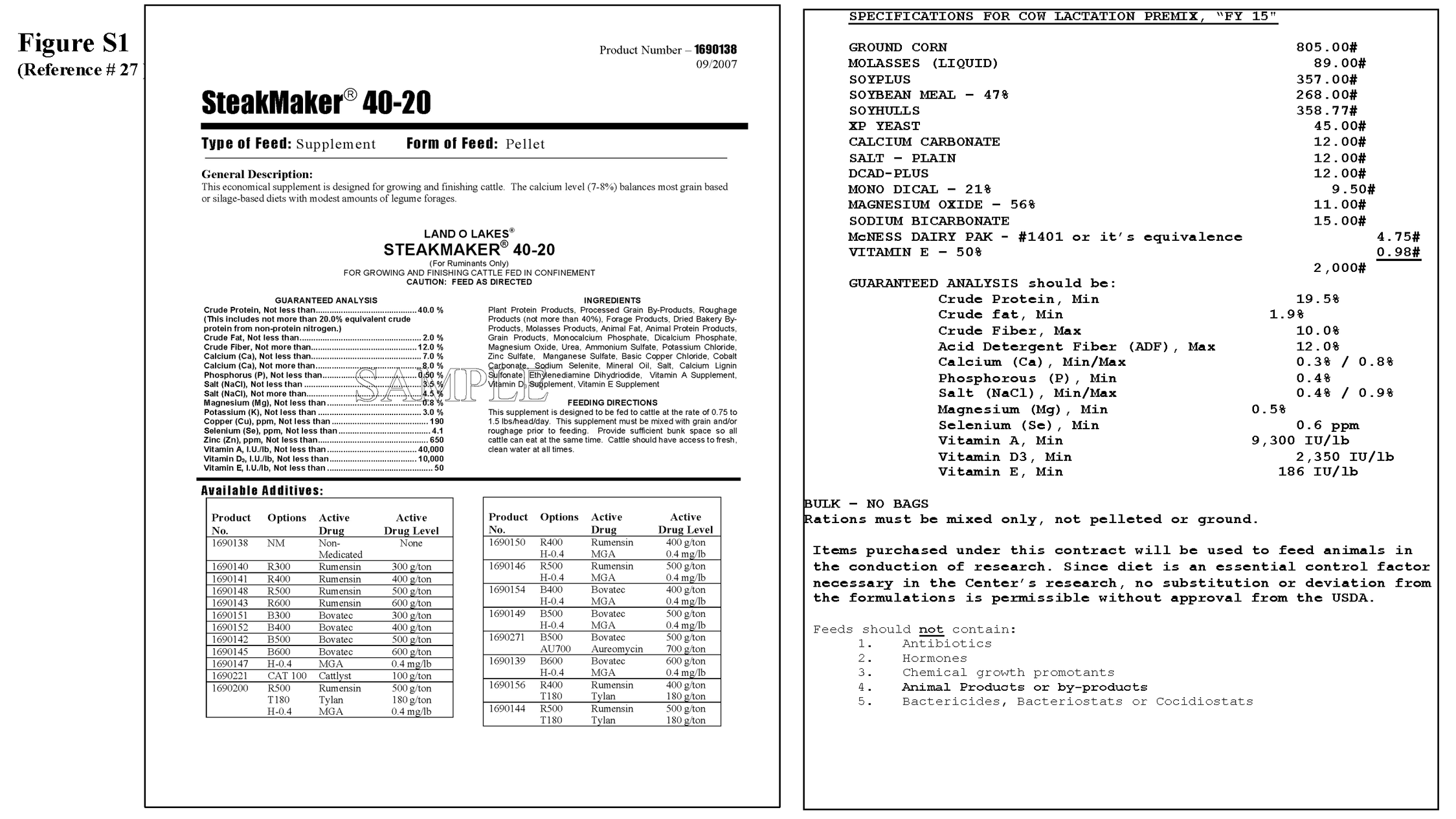

Supplement: S1 Figure — (TIF) [file pone.0313978.s003.tif]

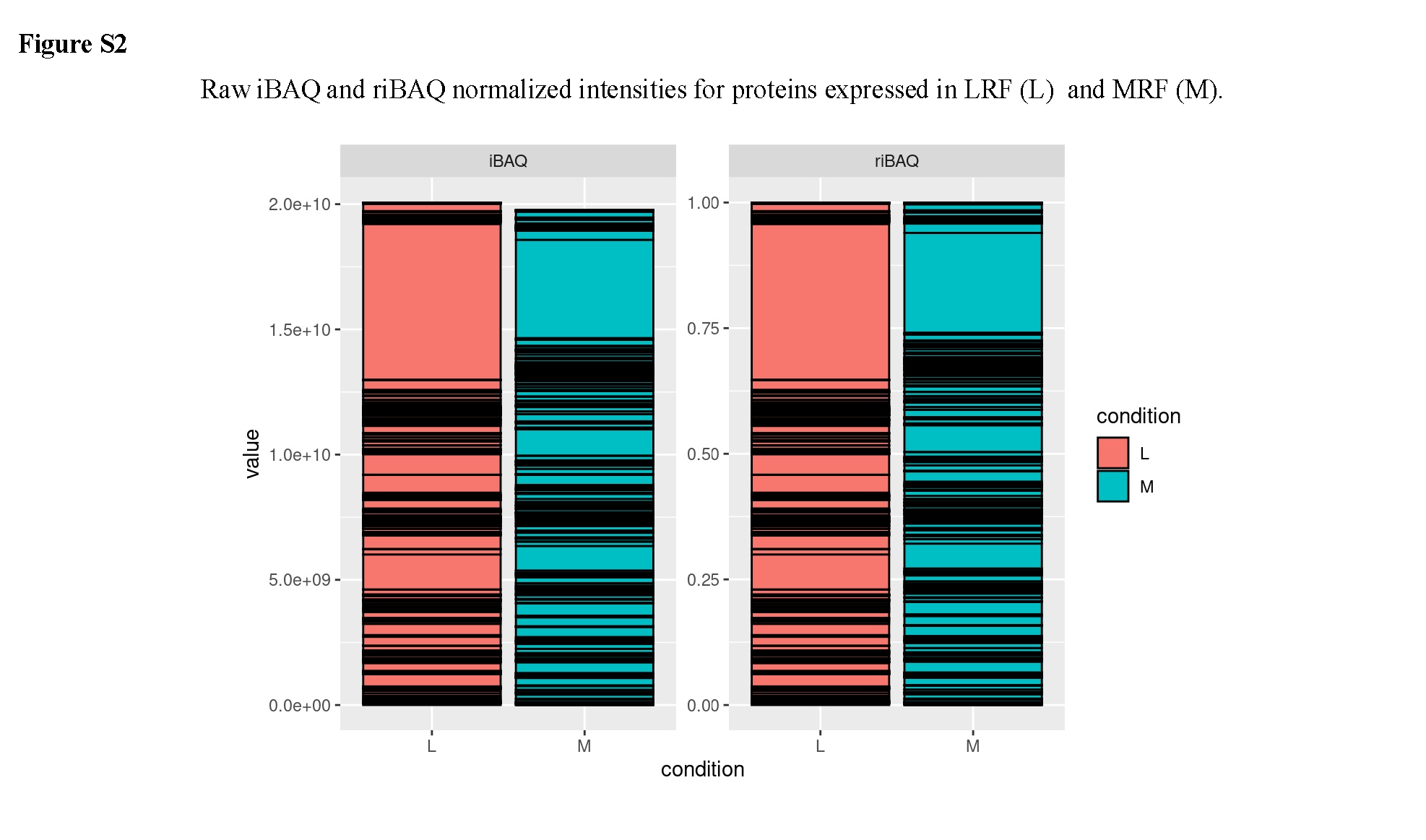

Supplement: S2 Figure — (TIF) [file pone.0313978.s004.tif]

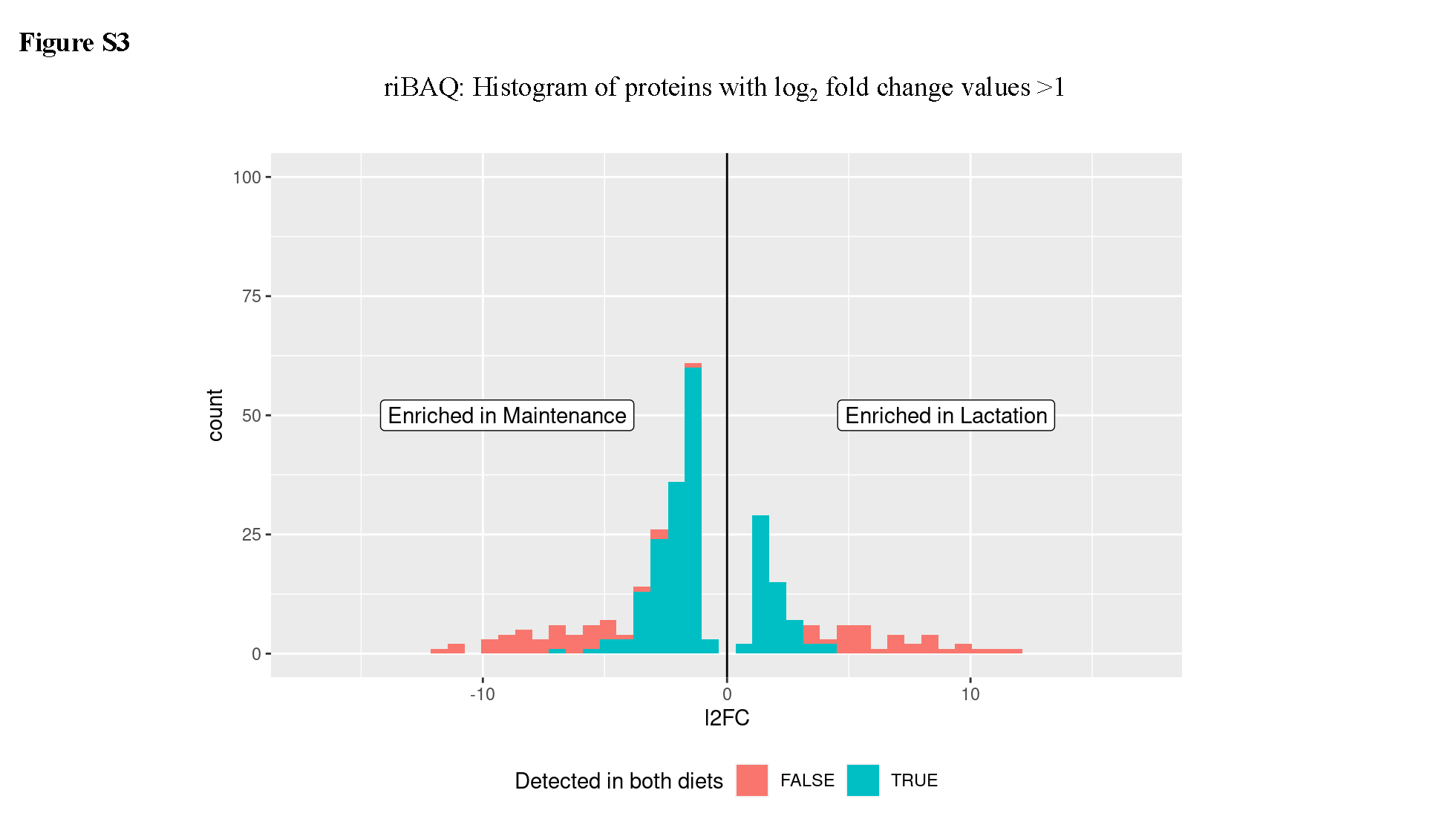

Supplement: S3 Figure — (TIF) [file pone.0313978.s005.tif]

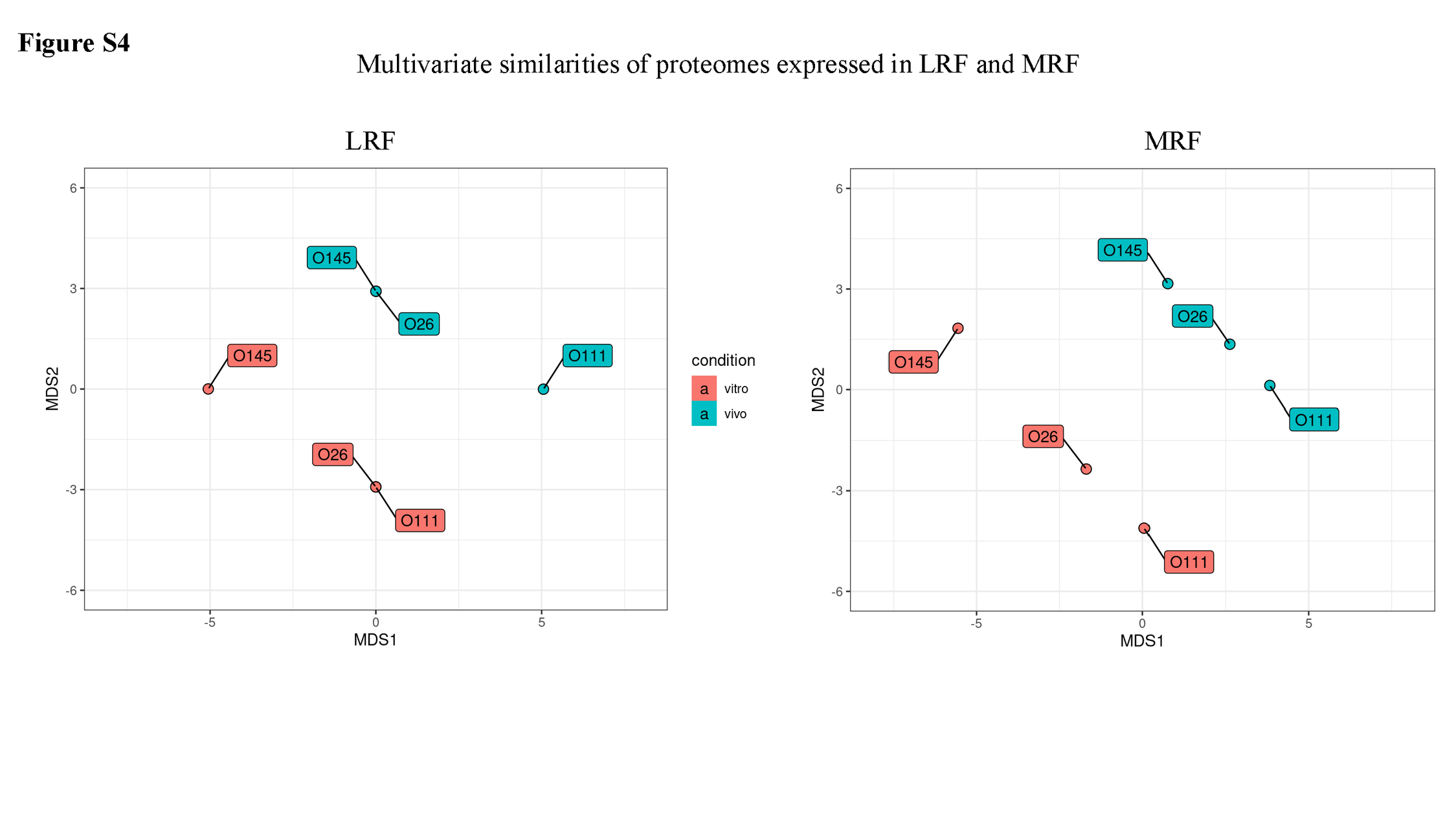

Supplement: S4 Figure — (TIF) [file pone.0313978.s006.tif]

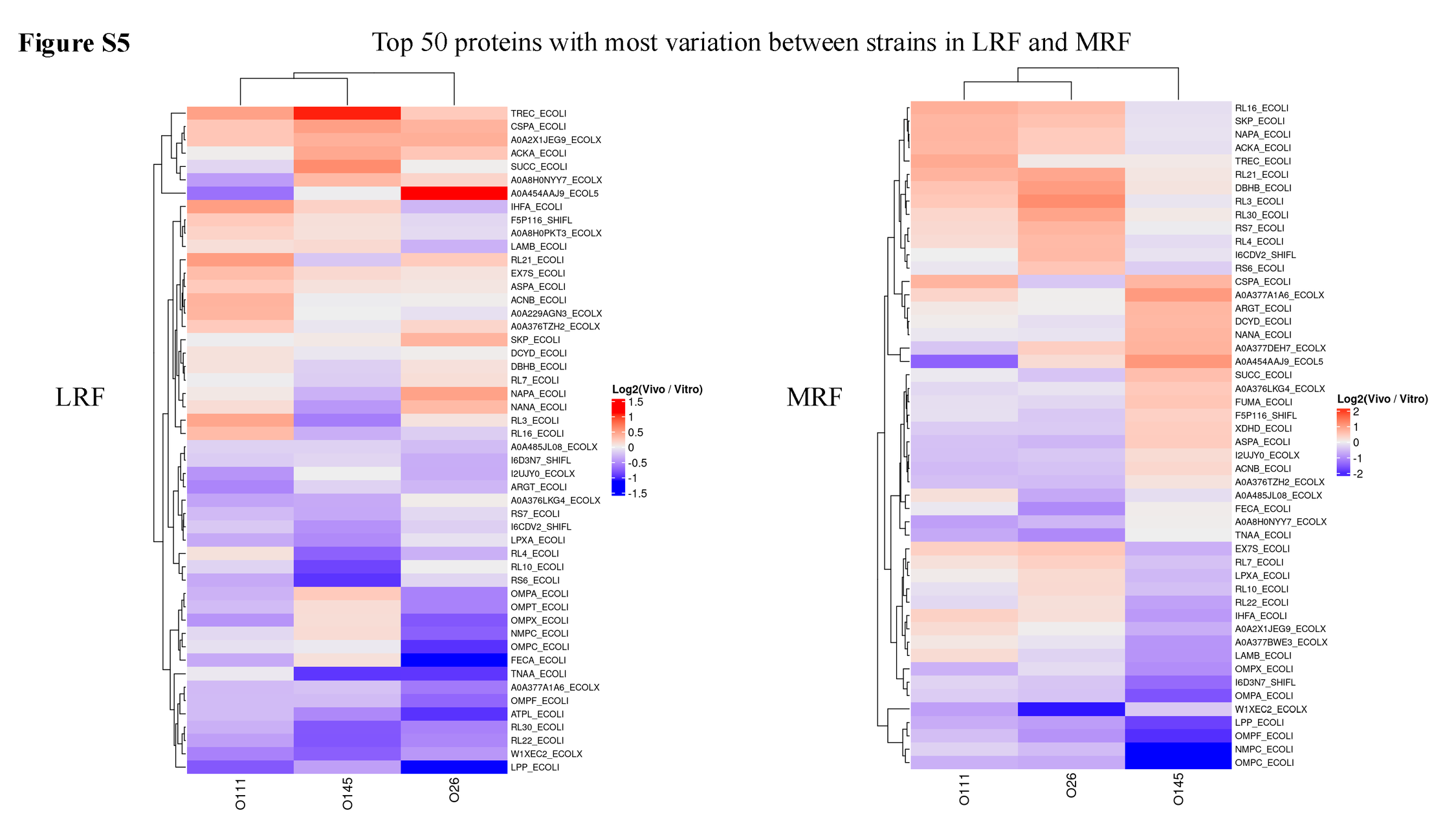

Supplement: S5 Figure — (TIF) [file pone.0313978.s007.tif]
