# Supplementary material for: Growth dynamics and protein-expression of Escherichia coli serotypes O26:H11, O111:H8 and O145:NM in the bovine rumen
Source: PLoS One. 2025 Jun 4;20(6):e0313978. doi: 10.1371/journal.pone.0313978 (PMC12136435; doi:10.1371/journal.pone.0313978)
Supplement: S1 Table — (DOCX) [file pone.0313978.s008.docx]

**Table S1. Recovery of bacteria from cartridges exposed to LRF in *in vitro* and *in vivo*.**

| **Bacterial strain** | | ***In vitro* in LRF** | ***In vivo* in LRF** |
| --- | --- | --- | --- |
|  |  | **Average^1^ Bacterial counts (cfu/ml)** | **Average Bacterial counts (cfu/ml)** |
| **O26:H11** | **0 h** | 5.2 ± 5 x 10^8^ | 1.35 ± 1.15 x 10^8^ |
|  | **48 h** | 4 ± 1 x 10^1^ | 3 ± 3 x 10^6^ |
| **O111:H8** | **0 h** | 5.76 ± 5 x 10^8^ | 6.3 ± 4 x 10^8^ |
|  | **48 h** | 1.25 ± 1.2 x 10^3^ | 8 ± 8 x 10^6^ |
| **O145:NM** | **0 h** | 3.25 ± 3 x 10^8^ | 1.25 ± 0.85 x 10^8^ |
|  | **48 h** | 6 ± 4 x 10^3^ | 1.07 ± 1 x 10^7^ |
| ***E. coli* Nal^R^ (NADC 5735)** | **0 h** | 4 ± 4 x 10^8^ | 7.5 ± 6.5 x 10^8^ |
|  | **48 h** | 2 ± 0.1 x 10^5^ | 5 ± 5 x 10^5^ |

^1^Average from two separate experiments.
