# Supplementary material for: Growth dynamics and protein-expression of Escherichia coli serotypes O26:H11, O111:H8 and O145:NM in the bovine rumen
Source: PLoS One. 2025 Jun 4;20(6):e0313978. doi: 10.1371/journal.pone.0313978 (PMC12136435; doi:10.1371/journal.pone.0313978)
Supplement: S2 Table — (DOCX) [file pone.0313978.s009.docx]

**Table S2. Recovery of bacteria from cartridges exposed to MRF in *in vitro* and *in vivo*.**

| **STEC strain** | | ***In vitro* in MRF** | ***In vivo* in MRF** |
| --- | --- | --- | --- |
|  |  | **Average^1^ Bacterial counts (cfu/ml)** | **Average Bacterial counts (cfu/ml)** |
| **O26:H11** | **0 h** | 2.63 ± 2 x 10^8^ | 5.55 ± 4 x 10^8^ |
|  | **48 h** | 3.5 ± 0.5 x 10^6^ | 1 ± 0 x 10^7^ |
| **O111:H8** | **0 h** | 1.4 ± 1.1 x 10^8^ | 1.54 ± 1.3 x 10^9^ |
|  | **48 h** | 7.6 ± 1.4 x 10^6^ | 2.2 ± 1.8 x 10^7^ |
| **O145:NM** | **0 h** | 8.2 ± 5.8 x 10^7^ | 2.61 ± 2 x 10^9^ |
|  | **48 h** | 5.45 ± 0.8 x 10^6^ | 2.64 ± 1.36 x 10^7^ |
| ***E. coli* Nal^R^ (NADC 5735)** | **0 h** | 3.9 ± 4 x 10^8^ | 6 ± 4 x 10^8^ |
|  | **48 h** | 1.9 ± 0.1 x 10^5^ | 1.4 ± 0.3 x 10^7^ |

^1^Average from two separate experiments.
